# Clinical and humanistic burden among pediatric patients with neurofibromatosis type 1 and plexiform neurofibroma in the USA

**DOI:** 10.1007/s00381-022-05513-8

**Published:** 2022-05-17

**Authors:** Xiaoqin Yang, Hyun Kyoo Yoo, Suvina Amin, Wendy Y. Cheng, Sanjana Sundaresan, Lujia Zhang, Mei Sheng Duh

**Affiliations:** 1 Merck & Co., Inc, Kenilworth, NJ USA; 2 AstraZeneca, Cambridge, UK; 3 AstraZeneca, Gaithersburg, MD USA; 4 Analysis Group, Inc, Boston, MA USA

**Keywords:** Neurofibromatosis type 1, Plexiform neurofibroma, Patient-reported outcomes, Debulking surgery, Health-related quality of life

## Abstract

**Purpose:**

To assess clinical and humanistic burden among pediatric patients with neurofibromatosis type 1 (NF1) and plexiform neurofibroma (PN) in the USA.

**Methods:**

NF1-PN patients aged 8–18 years (treatment-naïve or ≤ 1 month of selumetinib treatment) and their caregivers and caregivers of similar patients aged 2–7 years were recruited through the Children’s Tumor Foundation to participate in an online cross-sectional survey (December 2020–January 2021). Caregivers provided data on patients’ demographic and clinical characteristics and burden of debulking surgeries. Patients and caregivers provided self-reported or proxy responses to health-related quality of life (HRQoL) questions using validated instruments.

**Results:**

Sixty-one patients and 82 caregivers responded to the survey. Median (range) age of patients was 11.5 (3–18) years, and 53.7% were female. Most were treatment-naïve (97.6%), with NF1-PN diagnosis for > 5 years (68.3%). Most patients (59.8%) had > 1 PN and 11.0% reporting > 5 PNs. Common NF1-PN symptoms included pain (64.6%), disfigurement (32.9%), and motor dysfunction (28.0%). Patients and caregiver proxies reported low overall HRQoL and reduced physical, emotional, social, and school functioning. Patients also reported considerable pain severity, interference, daily activity impairments, and movement difficulty. Few patients had received complete resections of their tumors (12.2%). 39.0% reported ≥ 1 debulking surgery, among whom, 15.6% had complications, and debulking surgery-related hospitalizations were common (53.1%).

**Conclusions:**

The clinical and humanistic burden among pediatric NF1-PN patients is substantial. While debulking surgeries are used for symptom management, they are associated with considerable clinical sequelae. Results highlight a need for improved disease management strategies.

**Supplementary information:**

The online version contains supplementary material available at 10.1007/s00381-022-05513-8.

## Introduction

Neurofibromatosis (NF) is an autosomal dominant condition present in about one in 3,000 live births and is the most prevalent form of NF [1]. Common distinguishing features of NF1 include the development of neurofibromas, café au lait skin spots, and iris Lisch nodules [1]. In 2021, diagnostic criteria were updated and included the addition of choroidal abnormalities, updates to orthopedic and pigmentary finding criteria, and further clarification on the genetics and inheritance patterns of NF1 [2, 3]. In addition to the physical manifestations of NF1, many patients may experience learning disabilities (>50%) and cognitive impairments (≥1 impairments in 81% of cases) [1, 4].

NF1 is associated with a high risk of central and peripheral nervous system tumors, with the most common type being plexiform neurofibromas (PNs), which occur in up to 50% of NF1 patients [5–8]. Although PNs affect multiple cell types, their occurrence in neuronal cells is often accompanied by debilitating pain, motor impairments, and visual dysfunction [9–11]. PNs develop in regions of the body including the head, neck, and torso and may invade neighboring structures resulting in further pain, disfigurement, organ compression, and dysfunction [12–15]. Complete surgical resection or debulking surgery have been the mainstay of treatment to help restore appearance and functionality to an affected region [16]. However, PNs are often inoperable due to size, location, interdigitation into the surrounding tissue or nerve, and potential post-surgical complications including hematomas and delayed healing of the surgical site [17, 18]. PNs can transform into malignant peripheral nerve sheath tumors (MPNSTs) [19]. Patients with NF1 have a 8–13% risk of developing an MPNST with the risk increasing 20-fold if the patient has an internal PN. Five-year disease-specific survival for patients with NF1 and a diagnosis of MPNST is 54% [19].

On April 10, 2020, the US Food and Drug Administration (FDA) approved the use of selumetinib as the only treatment option among pediatric patients aged ≥ 2 years, with symptomatic inoperable PN caused by NF1. Selumetinib is a mitogen-activated protein kinase enzyme inhibitor that targets the RAS signaling pathway which is overactive in patients with NF1 [20]. It has shown clinical benefit resulting in the shrinkage of PNs as early as 4 months after treatment initiation, with responses lasting longer than a year in most patients [21].

Given the varied manifestations of and impairments associated with NF1-PNs, prior evidence has indicated that the humanistic burden (i.e., burden of a disease from the perspective of an individual) among pediatric patients with NF1-PN is substantial, with affected children reporting significantly worse overall health-related quality of life (HRQoL) and reduced social-emotional functioning relative to their peers in the general population [14, 22–24]. Moreover, pediatric patients who report physical pain have more severe symptoms along with worse motor, social, and emotional functioning, compared to those who do not [14]. Despite these observations, real-world evidence describing the clinical burden and HRQoL among pediatric patients with NF1-PN is scarce. Further, there is a lack of evidence regarding the burden of debulking surgeries among these patients. The present study aimed to characterize the clinical burden, HRQoL, and burden of debulking surgeries among pediatric NF1-PN patients in the USA.

**Table 1:** Health-related quality of life among pediatric patients with NF1-PN

	**Child self-reported response**	**Caregiver proxy-reported response**
**Overall HRQoL**		
**PedsQL acute version**	***N* = 61**	***N* = 82**
**Physical functioning**		
Mean (SD)	63.7 (25.1)	65.0 (24.3)
Median (range)	65.6 (0.0, 100.0)	68.8 (3.1, 100.0)
**Emotional functioning**		
Mean (SD)	56.1 (20.0)	54.9 (24.1)
Median (range)	55.0 (10.0, 100.0)	50.0 (0.0, 100.0)
**Social functioning**		
Mean (SD)	60.7 (22.6)	60.5 (26.1)
Median (range)	65.0 (0.0, 100.0)	62.5 (0.0, 100.0)
**School functioning**		
Mean (SD)	50.3 (22.9)	54.0 (24.4)
Median (range)	50.0 (5.0, 100.0)	55.0 (0.0, 100.0)
**Total**		
Mean (SD)	58.5 (19.3)	59.1 (20.6)
Median (range)	62.0 (15.2, 96.7)	60.9 (15.2, 100.0)
**EQ-5D-Y**	***N* = 61**	Not applicable
**Mobility, *n* (%)**		
No problems	47 (77.0)	
Some problems	13 (21.3)	
A lot of problems	1 (1.6)	
**Looking after myself, *n* (%)**		
No problems	46 (75.4)	
Some problems	13 (21.3)	
A lot of problems	2 (3.3)	
**Doing usual activities, *n* (%)**		
No problems	32 (52.5)	
Some problems	26 (42.6)	
A lot of problems	3 (4.9)	
**Having pain or discomfort, *n* (%)**		
No problems	21 (34.4)	
Some problems	29 (47.5)	
A lot of problems	11 (18.0)	
**Feeling worried, sad, or unhappy, *n* (%)**		
No problems	23 (37.7)	
Some problems	31 (50.8)	
A lot of problems	7 (11.5)	
**EQ-VAS**	***N* = 61**	
Mean (SD)	77.6 (18.0)	
Median (range)	82.0 (31.0, 100.0)	
**Physical functioning**		
**Reported difficulties with movement in the last 7 days**	***N* = 26**	***N* = 20**
**PROMIS—Mobility: T score**		
Mean (SD)	40.2 (8.6)	36.0 (5.5)
Median (range)	39.5 (33.0, 46.0)	35.0 (33.0, 40.5)
**PROMIS—Upper Extremity Functioning: T score**		
Mean (SD)	39.5 (13.5)	29.1 (7.8)
Median (range)	39.5 (32.0, 49.0)	29.0 (25.5, 34.0)
**Pain**		
**PII**		
**All patients**	***N* = 61**	***N* = 82**
Mean (SD)	1.5 (1.8)	1.3 (1.8)
Median (range)	0.0 (0.0, 5.3)	0.0 (0.0, 6.0)
**Reported pain in the last 7 days**	***N* = 31**	***N* = 39**
Mean (SD)	3.0 (1.5)	2.7 (1.8)
Median (range)	3.3 (0.0, 5.3)	2.7 (0.0, 6.0)
**Modified NRS-11**		Not applicable
**All patients**	***N* = 61**	
Mean (SD)	2.7 (3.2)	
Median (range)	1.0 (0.0, 10.0)	
No pain (0), *n* (%)	30 (49.2)	
Mild pain (1–3), *n* (%)	8 (13.1)	
Moderate pain (4–6), *n* (%)	12 (19.7)	
Severe pain (7–10), *n* (%)	11 (18.0)	
**Reported pain in the last 7 days**	***N* = 31**	
Mean (SD)	5.3 (2.4)	
Median (range)	6.0 (1.0, 10.0)	
Mild pain (1–3), *n* (%)	8 (25.8)	
Moderate pain (4–6), *n* (%)	12 (38.7)	
Severe pain (7–10), *n* (%)	11 (35.5)	

## Methods

### Study design and sample selection

This observational, cross-sectional study used a one-time survey to assess the clinical burden, HRQoL, and debulking surgery burden among pediatric NF1-PN patients. Pediatric patients aged 8–18 years with NF1-PN and their caregivers, as well as caregivers of patients aged 2–7 years with NF1-PN, participated in an online survey from December 1, 2020, to January 14, 2021. Participants were recruited via email through the NF Registry, a patient-centered database managed by the Children’s Tumor Foundation (CTF), a global foundation who supports research to expand awareness of NF and accelerate patient care.

Pediatric patients were eligible to participate in the survey if they met the age requirement (aged 8–18 years), were naïve or new to selumetinib treatment (defined as ≤ 1 month of use), residents of the USA, and able to read and write English. Caregivers for all patients with NF1-PN (aged 2–18 years) were required to be aged 18 or older, residents of the USA, and able to read and write English. The ≤ 1 month period allowed for treatment with selumetinib ensured that these patients were eligible for the only approved drug for NF1-PN, but unlikely to have experienced a response to selumetinib due to the short period of exposure at the time of enrollment. Pediatric patients were excluded from the study if they were previously treated with selumetinib but were no longer receiving active treatment, ever treated with off-label treatments for NF1-PN (i.e., treated with binimetinib, cobimetinib, mirdametinib, or trametinib), or pregnant.

### Measurements and outcomes

Study measures included patient demographics, clinical characteristics, overall HRQoL, physical functioning, pain-related outcomes, and the burden of debulking surgeries. Patients provided self-reported responses to survey questions on HRQoL, and caregivers provided proxy responses to the same questions. Caregivers responded to questions on patient demographic and clinical characteristics and burden of debulking surgeries for the “full pediatric patient sample” (i.e., patients aged 8–18 years along with their caregivers and patients aged 2–7 years for whom their caregivers participated alone) (Supplemental Table 1).

### HRQoL among pediatric patients

HRQoL was measured using instruments previously validated in pediatric populations and caregivers of pediatric populations for proxy report versions [25–27]. Overall HRQoL and separate summary scores were assessed using the 23-item Pediatric Quality of Life Inventory (PedsQL)—generic scale, acute version [25, 26]. Total score and separate summary scores range from 0 to 100, with higher score representing better HRQoL (mean values are typically > 80 among healthy individuals) [25]. HRQoL was further assessed using the EQ-5D-Y [27], a preference-based instrument.

Physical functioning was assessed using the PROMIS [28] subscales for mobility and upper extremity functioning (eight items each) only if patients and/or caregivers reported that they or their child, respectively, had experienced difficulty with movement in the past 7 days. PROMIS scores were converted to standardized T-scores (i.e., scores were standardized to the US general population) with mean ± standard deviation (SD) of 50 ± 10; higher scores indicate better physical functioning.

Pain-related outcomes were assessed among patients who had reported experiencing pain as a result of their NF1-PN in the past 7 days. The single-item-modified Numeric Rating Scale [29] (NRS-11; range: 0–10; higher = more pain) was used to assess pain, and the six-item Pain Interference Index (PII; range: 0–6; higher = more pain interference) [30] was used to assess the level of pain interference with daily activities.

### Burden of debulking surgeries

The burden of debulking surgeries was evaluated based on the number of debulking surgeries since diagnosis of NF1-PN, physician assessment of tumor inoperability, reasons for not receiving surgeries (if applicable), occurrence of surgery (yes/no), and the number of acute complications and chronic post-operative symptoms associated with each debulking surgery (among those with ≥ 1 debulking surgery). Debulking surgery-related healthcare resource utilization (HRU) was measured from the first debulking surgery and included hospitalization frequency (total number of hospitalizations due to debulking surgeries), length of hospital stay (length of hospital stay for all if ≤ 3 hospitalizations or the three most recent hospitalizations due to debulking surgeries), and emergency room visit frequency (total number of emergency room visits due to debulking surgeries).

### Statistical analyses

Descriptive summary measures were calculated for all outcomes of interest for the full pediatric patient sample. Continuous variables were summarized using means (SDs) and medians (range), and categorical variables were summarized using frequencies and proportions.

## Results

### Baseline demographic and clinical characteristics

Overall, 61 pediatric patients aged 8–18 years and their caregivers and 21 additional caregivers of patients aged 2–7 years (total of *n* = 82 caregivers) participated in the survey. Demographic and clinical characteristics of the full pediatric patient sample (*n* = 82) are shown in Supplemental Tables 2 and 3, respectively. The median (range) age of pediatric patients was 11.5 (3.0–18.0) years, and 53.7% were female. Most pediatric patients were White/Caucasian (85.4%) and were predominantly from the South and West regions (31.7% and 28.0%, respectively).

**Table 2:** Debulking surgery burden among pediatric patients with NF1-PN

**Debulking surgeries since diagnosis with NF1-PN, *n* (%)**	***N* = 82**
0	50 (61.0)
1	18 (22.0)
2	8 (9.8)
3	1 (1.2)
4	2 (2.4)
5	1 (1.2)
> 5	2 (2.4)
**Physician assessment of at least 1 tumor as inoperable, *n* (%)**	***N* = 70**
Yes	35 (50.0)
No	29 (41.4)
Don’t know/unsure	6 (8.6)
**Surgical complications associated with debulking surgeries, *n* (%)**	***N* = 32**
No	26 (81.3)
Yes	5 (15.6)
Don’t know/unsure	1 (3.1)
**Types of surgical complications, *n* (%)**^**a**^	***N* = 5**
Acute complications	3 (60.0)
Delayed healing	1 (20.0)
Bleeding/hematoma	1 (20.0)
Infection	0
Necrosis	0
Other	1 (20.0)
Post-operative symptoms	2 (40.0)
Functional impairment	1 (20.0)
Nerve damage	1 (20.0)
Other	1 (20.0)
**Reasons for not receiving surgeries, *n* (%)**^**a**^	***N* = 50**
PN was small and/or asymptomatic	26 (52.0)
Perceived risk	15 (30.0)
Patient preference	5 (10.0)
Cost	2 (4.0)
Scheduling difficulty	1 (2.0)
Other reason(s) provided by the physician	20 (40.0)

^a^Patients could be included in more than one category. Therefore, the sum of the percentages may exceed 100%

Most pediatric patients had NF1 and PN diagnoses for > 5 years (80.5% and 68.3%, respectively) and were selumetinib-naïve (97.6%). Over half of pediatric patients (58.5%) had > 20 café au lait spots. A vast majority of pediatric patients (71.9%) had 1–2 PNs, 11.0% had > 5 PNs, and were most frequently reported to be located on the back (40.2%) and the head (32.9%).

Commonly reported comorbidities included attention-deficit hyperactivity disorder (56.1%) followed by headaches (47.6%). Common NF1-PN symptoms reported included pain (64.6%), disfigurement (32.9%), and motor dysfunction (28.0%). Approximately one-third of patients were treated with surgery (32.9%) and pain relievers (31.7%).

### Health-related quality of life

Statistics on self-reported responses from patients (aged 8–18; *n* = 61) and proxy-reported responses from the caregivers of the full pediatric patient sample (*n* = 82) are presented in Table 1.

### PedsQL

Mean patient self-reported scores were 63.7 (SD = 25.1) for physical functioning, 56.1 (SD = 20.0) for emotional functioning, 60.7 (SD = 22.6) for social functioning, and 50.3 (SD = 22.9) for school functioning, with a mean total score of 58.5. Mean caregiver proxy-reported scores were 65.0 (SD = 24.3) for physical functioning, 54.9 (SD = 24.1) for emotional functioning, 60.5 (SD = 26.1) for social functioning, and 54.0 (SD = 24.4) for school functioning, with a mean total score of 59.1.

### EQ-5D-Y

More than half of pediatric patients reportedly experienced pain or discomfort (65.5%) and felt worried, sad, or unhappy (62.3%). About half of pediatric patients reported problems with doing usual activities (47.5%). Approximately one-quarter of pediatric patients reported problems with mobility and looking after themselves. The mean score on the EQ-VAS (range: 0–100; worst [0] to best [100] imaginable health) was 77.6 (SD = 18.0).

### PROMIS: mobility and upper extremity functioning

Among the 26 pediatric patients with self-reported movement difficulty in the past 7 days, mean scores were 40.2 (SD = 8.6) for mobility and 39.5 (SD = 13.5) for upper extremity functioning. Among the 20 caregivers who reported that their patients experienced movement difficulty, proxy-reported responses yielded mean scores of 36.0 (SD = 5.6) and 29.1 (SD = 7.8) for mobility and upper extremity functioning, respectively.

### PII

Among all patients (aged 8–18), the mean score on the PII was 1.5 (SD = 1.8), which was similar to the mean caregiver proxy-reported score (mean [SD] = 1.3 [1.8]). Among 50.8% of patients who reported experiencing pain in the past 7 days, PII score was markedly higher (mean [SD]: = 3.0 [1.5]) than among the total sample of patients (aged 8–18); this PII score was similar for caregivers (47.6%) who reported that their pediatric patient experienced pain in the past 7 days (mean [SD] = 2.7 [1.8]).

### NRS-11

The mean score on the NRS-11 was 2.7 (SD = 3.2) among all patients aged 8–18. Among 50.8% of patients who reported pain in the past 7 days, the mean score was 5.3 (SD = 2.4). Among this group of patients, 25.8% experienced mild pain (score of 1–3), 38.7% experienced moderate pain (score of 4–6), and 35.5% experienced severe pain (score of 7–10).

### Debulking surgery burden

**Fig. 1:**
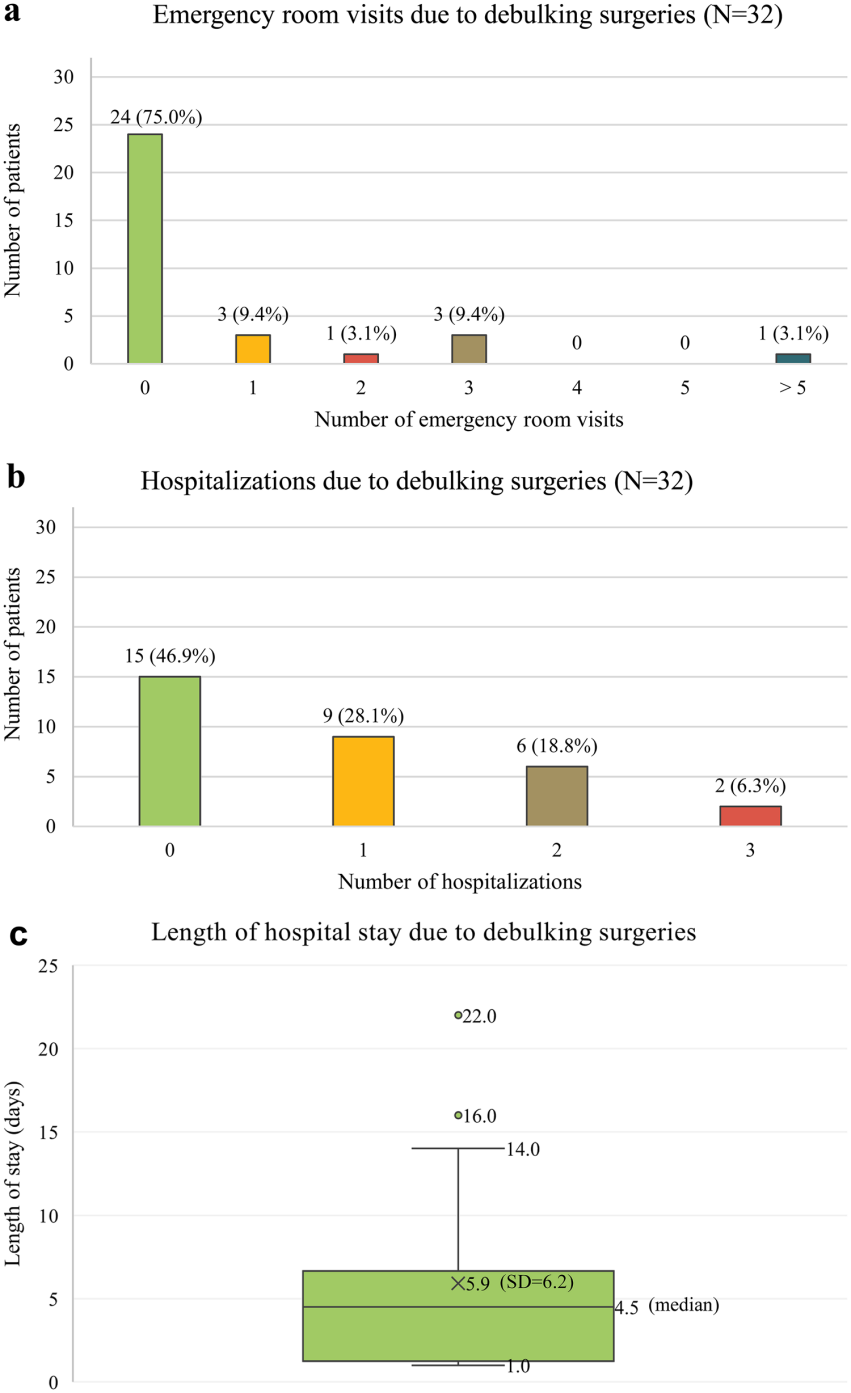
Various components of debulking surgery-related healthcare resource utilization among pediatric patients with NF1-PN are presented. **a** Emergency room visits: Assessments were made out of 32 patients who were reported as having received one or more debulking surgeries. **b** Hospitalizations: For patients with > 3 hospitalizations, the length of stay for the three most recent hospitalizations were captured. For patients with > 1 hospitalization, length of stay was averaged for a single value per patient. **c** Length of hospital stay: One outlying value (48 days) was set to missing

Few patients underwent complete resections of their tumors (12.2%; Supplemental Table 3), but 39.0% reported ≥ 1 debulking surgery (Table 2). Among a subset of 70 pediatric patients with available responses to a question about tumor inoperability, 50.0% were reported as having at least one inoperable tumor based on physician assessment. Among patients not treated with surgery, commonly reported reasons included small and/or asymptomatic PN (52.0%) and perceived risk (30.0%; Table 2). Among patients who received ≥ 1 debulking surgery, 15.6% were reported as having had complications. Among these patients, 60.0% experienced acute complications, including delayed healing (20.0%) and bleeding/hematoma (20.0%), and 40.0% experienced chronic post-operative symptoms, including functional impairment (20.0%) and nerve damage (20.0%; Table 2). Among 39.0% of patients who had received debulking surgeries, debulking surgery-related emergency room visits and hospitalizations were common (25.0% and 53.1%, respectively); the mean length of stay per hospitalization was 5.9 (SD = 6.2) days (Fig. 1).

## Discussion

This real-world study characterizes the clinical burden, HRQoL, and debulking surgery burden of NF1-PN among pediatric patients in the USA. The findings indicate that patients with NF1-PN experienced considerable pain and difficulty with movement. Moreover, their activities of daily living were impaired due to pain and motor dysfunction. Patients had severely diminished overall HRQoL and functional impairments across educational, emotional, social, and physical domains. Although debulking surgeries were used for symptomatic management, they were related to acute and chronic post-operative complications and high HRU. Furthermore, a considerable proportion of patients had inoperable PNs.

The present findings of low overall HRQoL and reduced functioning, particularly motor dysfunction as well as social and emotional functioning, are generally consistent with those of prior studies assessing the HRQoL of pediatric patients with NF1-PN using validated PRO instruments [14, 22–24, 31, 32]. Lai et al. [14] evaluated HRQoL among 140 pediatric patients with NF1-PN compared to US population norms using PROMIS modules and Neuro-QoL. Patients reported worse HRQoL than the norm in terms of anxiety, depressive symptoms, stigma, psychological stress experiences, meaning and purpose, mobility, peer relationships, positive affect and well-being, mobility, and upper extremity function. This growing body of evidence speaks to the multidimensional impact of NF1-PN on the lives of pediatric patients and the need for disease management through multidisciplinary teams including a physical therapist, occupational therapist, surgeon, psychiatrist/psychologist, pediatrician, or neurologist/neuro-oncologist.

Pain remains a major symptom of concern among pediatric NF1-PN patients. Approximately 65% of patients in the present study experienced pain (indicated by the EQ-5D-Y), corresponding to previous reports in this population [14]. Among patients with pain in the present study, 74.2% reported moderate to severe pain in the last 7 days based on the NRS-11. This high frequency and severity of pain are particularly concerning given that pain was identified as a major contributor to poor HRQoL among pediatric patients in prior studies [14, 22, 32]. Consistent with prior reports, the present study indicated considerable pain interference with daily activities despite the use of pain medication among 31.7% of patients [22, 23, 31]. In a prospective longitudinal study by Wolters et al. [23], pain interfered with the daily functioning of the majority of pediatric patients, with high rates of pain interference reported even among the 33% of patients who were regularly taking pain medications. Likewise, a prospective study of pediatric patients with NF1-PN enrolled in clinical trials reported considerable pain interference and diminished HRQoL, despite 42% of patients taking pain medication regularly [22, 31]. Thus, currently available pain medications appear to have limited effectiveness among this patient population, stressing the need for improved pain management strategies.

The present study fills an important knowledge gap by highlighting the substantial clinical burden and HRU associated with debulking surgeries in pediatric patients with NF1-PN. One prospective study of French NF1 patients (*n* = 201; age range: 7–84 years) reported that 47 (23%) had been hospitalized over 3 years, most commonly for the excision of multiple PNs (*n* = 51) or the treatment of malignant nerve sheath tumors (*n* = 21) [33]. A US-based retrospective chart review found that nearly half of pediatric patients with NF1 who had undergone PN surgery later experienced tumor progression, with a higher risk among younger children (> 10 years old) and children with tumors of the head/neck/face or tumors that could not be completely removed [34]. Thus, patients who undergo surgical resection may incur considerable risks and burden with limited long-term benefits, depending on prognostic factors. In the present study, the proportion of patients with inoperable PNs is likely a conservative underestimate of the true proportion. This is because some caregivers and their patients may not have been informed by their physicians regarding the PNs’ inoperability status and some caregivers may have been unfamiliar with the term “inoperable” and, thus, misinterpreted the question. As such, the 50% of patients with physician assessment of at least one tumor as inoperable likely represents the lower range of the true proportion of patients with inoperable PNs. Novel therapeutic interventions are urgently needed to combat the substantial clinical burden of NF1-PN among patients with inoperable tumors. In the Phase II SPRINT trial, selumetinib treatment led to durable tumor shrinkage and clinically meaningful improvements in HRQoL, suggesting that it may be a viable treatment option for this patient population [21].

The present study had several limitations. First, participants answered questions about their patient’s medical history without access to their medical records or clinician input, and, therefore, the responses to screening questions for eligibility and certain study outcomes may have been subject to recall bias. To mitigate this limitation, questions on patients’ length of hospitalizations related to debulking surgeries were restricted to the three most recent hospitalizations. Additionally, the acute version of the PedsQL was used due to its shorter recall period (relative to the standard version), which reduces measurement error [35]. Second, among the 32 patients with debulking surgeries, 17 were reported by their caregivers to have received only partial resections. The remaining 15 patients did not have partial resections reported by their caregivers, potentially due to respondent error (i.e., missing the option for “surgery” when asked about treatments received). Furthermore, two patients were reported as having partial resections but not debulking surgeries, potentially from not considering debulking surgeries the same as partial resections. Third, caregivers did not provide proxy responses to the EQ-5D-Y as the necessary version of the instrument was unavailable at the time of the study. Finally, the sample of participants may not be representative of all patients with NF1-PN in the USA as the sample was limited to patients (or caregivers) who were selumetinib-naïve or newly treated with selumetinib within the NF Registry and because there may have been referral bias as a result of recruiting participants from a single registry.

## Conclusions

This real-world study comprehensively assessed the clinical disease burden and HRQoL among pediatric patients with NF1-PN in the USA using validated patient-reported outcome instruments. The results indicate substantial clinical and humanistic burden of disease among patients, as evidenced by frequent pain and motor dysfunction, impairments in activities of daily living, and poor HRQoL. While debulking surgeries are used for symptom management, they were related to considerable clinical sequelae. Given the scarcity of data regarding the burden of illness and debulking surgeries among pediatric patients with NF1-PN, this study fills an important gap in the current literature and confirms the need for a multidisciplinary approach to treatment. This study also highlights the importance of collecting patient-centered outcomes that may enable healthcare teams to optimize disease management.

## Supplementary Information

Below is the link to the electronic supplementary material.

Supplementary file1 (DOCX 40 kb)

## Data Availability

The analytical datasets used in this study are available from the corresponding author on reasonable request.
