# Supplementary material for: Clinical and humanistic burden among pediatric patients with neurofibromatosis type 1 and plexiform neurofibroma in the USA
Source: Childs Nerv Syst. 2022 May 17;38(8):1513–22. doi: 10.1007/s00381-022-05513-8 (PMC9325812; doi:10.1007/s00381-022-05513-8)
Supplement: Supplementary file 1 — Supplementary file1 (DOCX 40 kb) [file 381_2022_5513_MOESM1_ESM.docx]

**SUPPLEMENTAL MATERIALS**

***Supplemental Table 1: Summary of Survey Components and their Respective Respondents***

| **Component** | **Respondent** |
| --- | --- |
| **Pediatric patient demographic and clinical characteristics** | Caregivers |
| **HRQoL among pediatric patients** |  |
| *Overall HRQoL* |  |
| Pediatric Quality of Life (PedsQL™) Acute Version   - Teen Report - Child Report - Parent Report for Teens - Parent Report for Children - Parent Report for Young Children - Parent Report for Toddlers | Patients aged 13-18  Patients aged 8-12  Caregivers of patients aged 13-18  Caregivers of patients aged 8-12  Caregivers of patients aged 5-7  Caregivers of patients aged 2-4 |
| EQ-5D   - EQ-5D-Y | Patients aged 8-18 |
| *Physical Functioning* |  |
| Patient-Reported Outcomes Measurement System (PROMIS®) Physical Function Short Forms- Mobility and Upper Extremity   - Pediatric Mobility - Pediatric Mobility* - Pediatric Upper Extremity - Pediatric Upper Extremity* - Parent Proxy Mobility - Parent Proxy Mobility* - Parent Proxy Upper Extremity - Parent Proxy Upper Extremity* | Patients aged 8-17  Patients aged 18  Patients aged 8-17  Patients aged 18  Caregivers of patients aged 5-17  Caregivers of patients aged 2-4, 18  Caregivers of patients aged 5-17  Caregivers of patients aged 2-4, 18 |
| *Pain* |  |
| Pain Interference Index (PII)   - PII - PII - Proxy Report - PII - Proxy Report* | Patients aged 8-18  Caregivers of patients aged 6-18  Caregivers of patients aged 2-5 |
| Modified Numerical Rating System (NRS-11)   - Modified NRS-11 | Patients aged 8-18 |
| **Burden of debulking surgeries** | Caregivers |

***** This instrument had not been validated among the specified population at the time of survey administration.

***Supplemental Table 2. Demographic Characteristics among Pediatric Patients with NF1-PN***

| **Treatment experience, n (%)** | **N = 82** |
| --- | --- |
| Selumetinib treatment naïve | 80 (97.6) |
| New user of selumetinib (≤ 1 month of use) | 2 (2.4) |
| **Age (years)** | **N = 82** |
| Mean (SD) | 11.5 (4.0) |
| Median (range) | 11.5 (3.0, 18.0) |
| **Sex, n (%)** | **N = 82** |
| Female | 44 (53.7) |
| Male | 38 (46.3) |
| **Race/Ethnicity, n (%)^a^** | **N = 82** |
| White or Caucasian | 70 (85.4) |
| Hispanic, Latino, or of Spanish origin | 16 (19.5) |
| Asian or Pacific Islander | 9 (11.0) |
| Black or African American | 7 (8.5) |
| American Indian or Alaska Native | 2 (2.4) |
| **Geographical region, n (%)** | **N = 82** |
| South | 26 (31.7) |
| West | 23 (28.0) |
| Midwest | 19 (23.2) |
| Northeast | 14 (17.1) |
| **Type of classroom setting, n (%)** | **N = 82** |
| Mainstream classroom without an individualized education plan (IEP) within a regular school | 40 (48.8) |
| Mainstream classroom with an IEP within a regular school | 15 (18.3) |
| Home school | 15 (18.3) |
| Special education classroom within a regular school | 8 (9.8) |
| Special education school | 1 (1.2) |
| Not applicable; child is not school aged | 3 (3.7) |
| **Type of learning model, n (%)^b^** | **N = 64** |
| Fully remote/online learning | 28 (43.8) |
| Hybrid learning (i.e., in-person and remote/online learning) | 19 (29.7) |
| Fully in-person learning | 16 (25.0) |
| Other | 1 (1.6) |
| **Learning or attention problems in school, n (%)^b^** | **N = 79** |
| Attention and learning problems | 45 (57.0) |
| Attention problems only | 14 (17.7) |
| Learning problems only | 4 (5.1) |
| Neither learning nor attention problems | 15 (19.0) |
| Unsure | 1 (1.3) |

**Notes:**

1. Patients could be included in more than one category. The sum of the percentages may exceed 100%.
2. Assessments were made among school-aged patients.

***Supplemental Table 3. Clinical Characteristics among Pediatric Patients with NF1-PN***

| **Time since NF1 diagnosis, n (%)** | **N = 82** |
| --- | --- |
| 0 to 5 years | 16 (19.5) |
| > 5 to 10 years | 28 (34.1) |
| > 10 to 15 years | 26 (31.7) |
| > 15 to 18 years | 12 (14.6) |
| **Time since PN diagnosis, n (%)** | **N = 82** |
| 0 to 5 years | 26 (31.7) |
| > 5 to 10 years | 34 (41.5) |
| > 10 to 15 years | 18 (22.0) |
| > 15 to 18 years | 4 (4.9) |
| **Number of café-au-lait spots, n (%)** | **N = 82** |
| 0 | 1 (1.2) |
| 1 to 5 | 1 (1.2) |
| 6 to 20 | 31 (37.8) |
| > 20 | 48 (58.5) |
| Unsure | 1 (1.2) |
| **Number of PNs, n (%)** | **N = 82** |
| 1 | 33 (40.2) |
| 2 | 26 (31.7) |
| 3 | 8 (9.8) |
| 4 | 4 (4.9) |
| 5 | 2 (2.4) |
| > 5 | 9 (11.0) |
| **Location of PNs, n (%)^a^** | **N = 82** |
| Back | 33 (40.2) |
| Head | 27 (32.9) |
| Spine | 24 (29.3) |
| Neck | 23 (28.0) |
| Abdomen | 23 (28.0) |
| Legs (right, left, or both) | 21 (25.6) |
| Chest | 21 (25.6) |
| Arms (right, left, or both) | 19 (23.2) |
| Unsure | 1 (1.2) |
| **Symptoms of NF1-PN, n (%)^a^** | **N = 82** |
| Pain | 53 (64.6) |
| Disfigurement | 27 (32.9) |
| Motor dysfunction | 23 (28.0) |
| Vision loss | 13 (15.9) |
| Bowel or bladder dysfunction | 11 (13.4) |
| Airway obstruction | 4 (4.9) |
| Other | 20 (24.4) |
| None of the above | 11 (13.4) |
| **Treatment received, n (%)^a^** | **N = 82** |
| Surgery | 27 (32.9) |
| Partial resection | 19 (23.2) |
| Complete resection | 10 (12.2) |
| Pain relievers | 26 (31.7) |
| Oncology medication^b^ | 8 (9.8) |
| Radiation therapy | 2 (2.4) |
| None of the above | 35 (42.7) |
| **Comorbid conditions, n (%)^a^** | **N = 82** |
| Attention-deficit hyperactivity disorder | 46 (56.1) |
| Headaches | 39 (47.6) |
| Autism | 15 (18.3) |
| Hypertension | 11 (13.4) |
| Epilepsy | 8 (9.8) |
| Vasculopathy | 3 (3.7) |
| Congenital heart disease | 1 (1.2) |
| None of the above | 14 (17.1) |

**Notes:**

1. Patients could be included in more than one category. Therefore, the sum of the percentages may exceed 100%.
2. Oncology medication included cabozantinib, dabrafenib, imatinib, and vemurafenib.
